# Genetic diversity in the transmission-blocking vaccine candidate *Plasmodium vivax* gametocyte protein Pvs230 from the China–Myanmar border area and central Myanmar

**DOI:** 10.1186/s13071-022-05523-0

**Published:** 2022-10-17

**Authors:** Xin Zhao, Yubing Hu, Yan Zhao, Lin Wang, Zifang Wu, Myat Thu Soe, Myat Phone Kyaw, Liwang Cui, Xiaotong Zhu, Yaming Cao

**Affiliations:** 1 Department of Immunology, College of Basic Medical Science, China Medical University, Shenyang, 110122 Liaoning People’s Republic of China; 2 Central Laboratory of the First Affiliated Hospital of China Medical University, Shenyang, China; 3 Myanmar Health Network Organization, Yangon, Myanmar; 4 Department of Internal Medicine, Morsani College of Medicine, University of South Florida, 3720 Spectrum Boulevard, Suite 304, Tampa, FL 33612 USA

**Keywords:** *Plasmodium vivax*, *Pvs230*, Gamete, Transmission-blocking vaccine, Genetic diversity

## Abstract

**Background:**

Sexual stage surface antigens are potential targets of transmission-blocking vaccines (TBVs). The gametocyte and gamete surface antigen P230, a leading TBV candidate, is critical for red blood cell binding during exflagellation and subsequent oocyst development. Here, the genetic diversity of *Pvs230* was studied in *Plasmodium vivax* parasite isolates from the China–Myanmar border (CMB) and central Myanmar.

**Methods:**

*Plasmodium vivax* isolates were collected in clinics from malaria-endemic areas of the CMB (143 samples) and Myanmar (23 samples). The interspecies variable part (IVP, nucleotides 1–807) and interspecies conserved part (ICP, 808–2862) of *Pvs230* were amplified by PCR and sequenced. Molecular evolution studies were conducted to evaluate the genetic diversity, signature of selection, population differentiation, haplotype network, and population structure of the study parasite populations and publicly available *Pvs230* sequences from six global *P. vivax* populations.

**Results:**

Limited genetic diversity was observed for the CMB (*π* = 0.002) and Myanmar (*π* = 0.001) isolates. Most amino acid substitutions were located in the IVP and cysteine-rich domain of *Pvs230*. Evidence of positive selection was observed for IVP and purifying selection for ICP. Codon-based tests identified specific codons under natural selection in both IVP and ICP. The fixation index (*F*_ST_) showed low genetic differentiation between East and Southeast Asian populations, with *F*_ST_ ranging from 0.018 to 0.119. The highest *F*_ST_ value (*F*_ST_ = 0.503) was detected between the Turkey and Papua New Guinea populations. A total of 92 haplotypes were identified in global isolates, with the major haplotypes 2 and 9 being the most abundant and circulating in East and Southeast Asia populations. Several detected non-synonymous substitutions were mapped in the predicted structure and B-cell epitopes of Pvs230.

**Conclusions:**

We detected low levels of genetic diversity of *Pvs230* in global *P. vivax* populations. Geographically specific haplotypes were identified for *Pvs230*. Some mutations are located within a potential B-cell epitope region and need to be considered in future TBV designs.

**Graphical Abstract:**

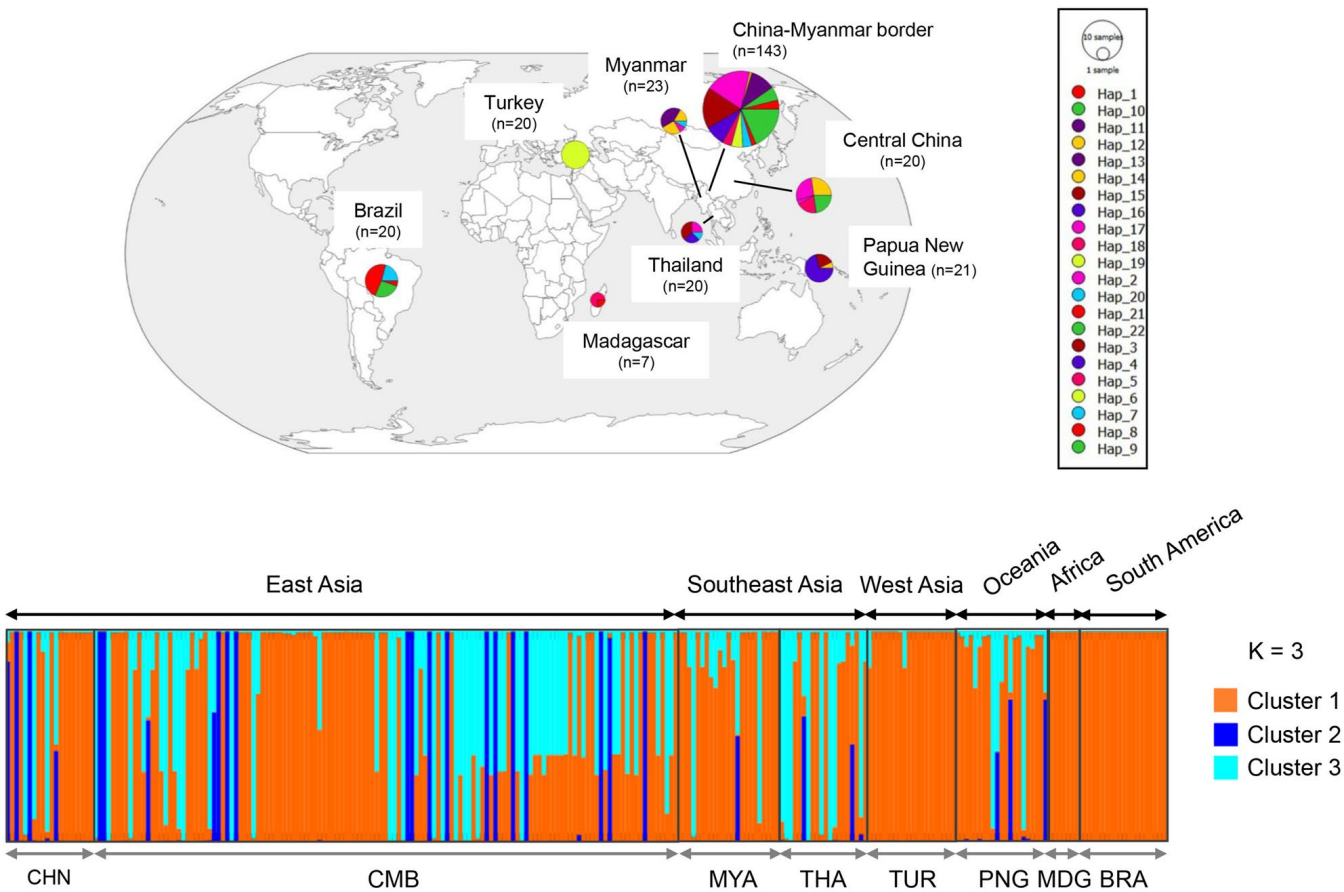

**Supplementary Information:**

The online version contains supplementary material available at 10.1186/s13071-022-05523-0.

## Background

The National Malaria Control Programs (NMCPs) in the Greater Mekong Subregion (GMS) have made substantial strides in malaria control, and in the past 9 years, most of the GMS countries endemic for malaria are making progress in reducing their malaria burden [1]. However, this success is faltering in some areas because of multidrug-resistant parasites, cross-border human migration, and the epidemics of the novel coronavirus disease 2019 (COVID-19) [2, 3, 4, 5]. Therefore, to ensure the most effective outcomes, the NMCPs in the GMS countries should design and adopt new strategies to eliminate malaria. The development of a highly effective malaria vaccine is an essential component of malaria control and eventual eradication. Transmission-blocking vaccines (TBVs) that reduce parasite transmission from humans to mosquitoes would be an important component toward the goal of malaria elimination.

TBVs induce antibodies that target the malaria antigens expressed in sexual stages (gametocyte, gamete, zygote, and ookinete), and are responsible for reducing the oocyst density in the mosquito midgut [6]. Based on the expression profile of target antigens, TBV candidates can be categorized into pre- (before zygote formation) and post-fertilization antigens. The expression of post-fertilization antigens, such as the zygote and ookinete surface antigens, P25/28, is typically limited to the mosquito stage of the parasite life cycle [7]. P25 and P28 are effective for inducing transmission-blocking activity (TBA), and have exhibited a transmission-blocking effect in phase I clinical trials, albeit with some adverse events [8]. Post-fertilization antigens are not expressed in blood-stage parasites and hence are not exposed to the human immune response by natural malaria infections. In contrast, pre-fertilization antigens P230 and P48/45, which belong to a family of 6-cysteine-containing domain (6-cys) proteins, are both found on the surface of gametocytes and gametes during infection, and therefore are exposed to the host immune system [9]. Pfs230 is a 363-kDa protein containing 14 cysteine-motif (CM) domains and elicits a transmission-reducing humoral immune response during natural malaria infection [10, 11, 12]. In Pfs230-disrupted parasites, the exflagellation centers, oocyst intensity, and mosquito infectivity are significantly reduced [13]. In standard membrane-feeding assays (SMFA), monoclonal antibodies against Pfs230 exhibited strong transmission-reducing activity (TRA) [14, 15]. Antibodies raised against both the N-terminal pro-domain (443–558 amino acids) and CM domains (443–1132 aa) and the CM1 domains (589–730 aa) of the Pfs230 protein exhibited efficient TBA [16, 17, 18]. Collectively, these results indicate that Pfs230 is a promising target for TBV.

A 6-cys domain protein in *Plasmodium vivax* has been identified as an orthologue of P230 in *Plasmodium falciparum*. Although Pfs230 has been studied extensively, limited studies have focused on Pvs230. Genetic diversity studies for vaccine candidate genes in malaria-endemic areas will provide useful information for vaccine design. Studies on asexual-stage vaccine candidate genes, such as *ama1*, revealed that the polymorphic antigen-induced immune response is allelic variant-dependent, which will limit the efficacy of the vaccine design based on this antigen [19, 20]. To date, no genetic diversity data exist for *Pvs230* in the China–Myanmar border (CMB) region and Myanmar, where *P. vivax* transmission persists. Thus, in the present study, the polymorphisms, neutral selections, genetic differentiation, haplotype network, and population structure of *Pvs230* were analyzed in *P. vivax* populations from this region. These analyses revealed limited genetic diversity in the *Pv230* gene. Neutrality tests identified *Pvs230* domains that are under positive and purifying selection. Although *Pvs230* haplotypes seem to be continent-specific, little genetic differentiation was found among East and Southeast Asia populations. The same major haplotypes were circulating in these endemic areas, which may facilitate TBV design for controlling malaria in these regions.

## Methods

### Sample collection and ethics

A total of 166 blood samples were collected by finger-pricking between April 2011 and October 2012 in the CMB region [21]. Twenty-three samples were collected near Mandalay in Myanmar in 2015. *Plasmodium vivax* infections were determined by either rapid diagnostic test (RDT) or microscopic examination of Giemsa-stained thick smears. All research involving human subjects in this study received ethical approval from the Health Department of Kachin in Myanmar and the institutional ethics committees of China Medical University, China. All volunteers gave written informed consent to participate in the study and provide blood samples.

### DNA extraction, polymerase chain reaction (PCR), and sequencing of the *Pvs230* gene

Genomic DNA was extracted from the collected *P. vivax* samples using the TIANamp Genomic DNA kit (TIANGEN, Beijing, China). The interspecies variable part (IVP, corresponding to nucleotides [nt] 1–807) and the interspecies conserved part (ICP, nt 808–2862) of the *Pvs230* gene, based on the Sal-I reference (PVX_003905 in PlasmoDB), were amplified by two independent PCRs using two sets of primers (Additional file 1: Table S1). The 20 μl PCRs consisted of 1× KOD-Plus-Neo buffer, 200 μM dNTPs, 1 mM MgSO_4_, 0.25 µM each specific forward and reverse primer, 0.4 units of KOD-Plus-Neo DNA polymerase (Toyobo, Osaka, Japan), and 1 μl of sample DNA. The reaction was run at 94 °C for 5 min, followed by 45 cycles of 94 °C for 30 s, 55 °C for 15 s, and 68 °C for 3 min, with a final extension at 68 °C for 5 min. The PCR products were purified using the ExoSAP-IT reagent (Thermo Fisher, MA, USA) and subjected to Sanger sequencing (BGI Science, Beijing, China) on both strands with primers listed in Additional file 1: Table S1 using an ABI Prism^®^ BigDye™ cycle sequencing kit (Applied Biosystems, CA, USA) on an ABI 3730XL DNA analyzer.

### Sequence assembling and polymorphism analysis

Raw sequence data of *Pvs230* were assembled, edited, and aligned to the *P. vivax* Sal-I reference sequence using the CLUSTALW program in MEGA 7.0 [22]. A 2811-base-pair (bp) region (nt 1–2862) encompassing the IVP and ICP of *Pvs230* (containing cysteine-rich domain [CRD]I–IV) was assembled, excluding the primer sequences. To investigate the global population structure of *Pvs230*, 108 publicly available *Pvs230* sequences were retrieved from GenBank, representing six locations: Papua New Guinea (PNG) (AB574595–AB574615), Thailand (AB574575–AB574594), Central China (Hubei province, AB574574–AB574555), Madagascar (AB574548–AB574554), Turkey (AB574528–AB574547), and Brazil (AB574508–AB574527) [23]. Molecular evolution analysis of the *Pvs230* gene, including the number of polymorphic sites (*S*), the total number of mutations (*η*), the average number of nucleotide differences (*k*), nucleotide diversity (*π*), the number of haplotypes (*H*), haplotype diversity (Hd), and the number of synonymous mutations (SP) and non-synonymous mutations (NS), was performed using DnaSP, version 6.12.03 [24]. Sliding window plots of *π* values across the IVP and ICP of *Pvs230* were generated using a 90-bp sliding window with 3-bp step size [24]. Sequences generated in this study were submitted to GenBank (accession numbers OP429256–OP429421).

### Statistical analysis

To test the neutral theory of evolution in the *Pvs230* gene, *d*_N_–*d*_S_, Tajima’s *D*, Fu and Li’s D* and F*, and McDonald–Kreitman (MK) indices were calculated using DnaSP v6.12.03 [24]. The rates of synonymous (*d*_S_) and non-synonymous (*d*_N_) mutations were estimated and compared by the *Z*-test (*P* < 0.05) in MEGA 7 using the Nei–Gojobori method with Jukes and Cantor correction [22]. A value of *d*_N_–*d*_S_ > 0 implies a positive selection, whereas *d*_N_–*d*_S_ < 0 designates a purifying selection [25]. In Tajima’s *D* test, the departure from neutrality in the nucleotide frequency distributions was determined by comparing the values of *θ* (estimated nucleotide diversity) derived from *π* and *S* [26]. Fu and Li’s D* and F* tests were used to test the hypothesis that all mutations are selectively neutral, and the mutations in the external branches were compared to the genealogy branches [27]. Positive values of Tajima’s *D* and Fu and Li’s D* and F* tests correspond to positive diversifying selection or population structuring due to an excess of variants at intermediate frequencies, whereas a negative value indicates population size expansion and/or negative/purifying selection. Sliding window plots with a window size of 90 and a step size of 3 were also generated for the Tajima’s *D* and Fu and Li’s D* and F* values, and *d*_N_–*d*_S_ was used to identify regions of *Pvs230* under selection [28]. The sequence of *Plasmodium cynomolgi* P230 (GenBank accession no. AB574620) was used as the interspecies outgroup for the MK test, which allows for determination of the ratio of synonymous substitutions to non-synonymous substitutions between and within species [29]. An excess of the ratio between species versus within species suggests purifying selection. A two-tailed Fisher’s exact test was computed to determine statistical significance (*P* < 0.05). A codon-based test using SLAC (Single-Likelihood Ancestor Counting) [30] and FUBAR (Fast, Unconstrained Bayesian AppRoximation) [31] methods implemented in the Datamonkey web server [32] was used to identify the existence of positive selection pressure on individual amino acids of the protein. Sites were considered under positive selection if the *d*_N_–*d*_S_ indicated high statistical significance (*P* < 0.05).

### *F*_ST_, haplotype network construction, and phylogenetic analysis

The degree of genetic differentiation in *Pvs230* among global isolates was estimated by calculating Wright’s fixation index (*F*_ST_) using Arlequin software, version 3.5.2.2 [33]. Interpretation of *F*_ST_ values is defined as described previously [34], with no differentiation (0), low genetic differentiation (≤ 0.15), moderate genetic differentiation (0.15–0.25), and high differentiation (≥ 0.25). A *P*-value < 0.05 was considered significant difference. To investigate the genetic relatedness among the *Pvs230* haplotypes, a haplotype network was constructed using the median-joining (MJ) method implemented in the PopART v1.7 software with a haplotype frequency > 1 [35]. A phylogenetic tree of *Pvs230* sequences (nt 52–2862) from the global isolates [36] was constructed by the NJ method [37] with a bootstrap of 1000 replicates using MEGA 7.0 software [22]. Final trees were drawn and edited using iTOL version 6 (https://itol.embl.de/).

### Population structure

Population structure and clusters of global isolates based on *Pvs230* haplotypes were investigated using the Bayesian model-based clustering method implemented in STRUCTURE software, version 2.3.4 [38]. Ten independent runs were performed with values of assumed clusters (*K*) ranging from 1 to 10. Each run was carried out with a “burn-in” period of 10^5^ and Markov chain Monte Carlo (MCMC) length of 2 × 10^5^ iterations under an admixture model with correlated allele frequencies among populations. The most probable *K* was inferred by the Evanno et al. method [39] using the online Structure Harvester v0.6.94 [40]. CLUMPP software [41] was used to address label switching within the determined value of *K*, using the greedy algorithm to increase speed, and a run of 1000 random repeats of the data. The graphical depiction of the STRUCTURE results was generated using Distruct software [42].

### Prediction of B-cell epitopes concerning single-nucleotide polymorphisms (SNPs) in IVP and ICP of *Pvs230*

The B-cell epitopes in IVP and ICP of *Pvs230* were predicted using the ABCpred server (www.imtech.res.in/raghava/abcpred) with a threshold of 0.7 and a window length of 16 [43], and using BepiPred-2.0 (https://services.healthtech.dtu.dk/service.php?BepiPred-2.0) with a threshold of 0.5 and peptide length > 10 [44]. The three-dimensional (3D) structure of *Pvs230* protein was predicted using the I-TASSER meta-threading procedure (https://zhanglab.ccmb.med.umich.edu/I-TASSER/) [45]. The overlapped regions in the B-cell epitopes from the prediction software and the mutation sites were mapped on a 3D structure of *Pvs230* using PyMOL software (http://www.scalacs.org/TeacherResources).

## Results

### Sequence polymorphism in *Pvs230* gene

**Fig. 1:**
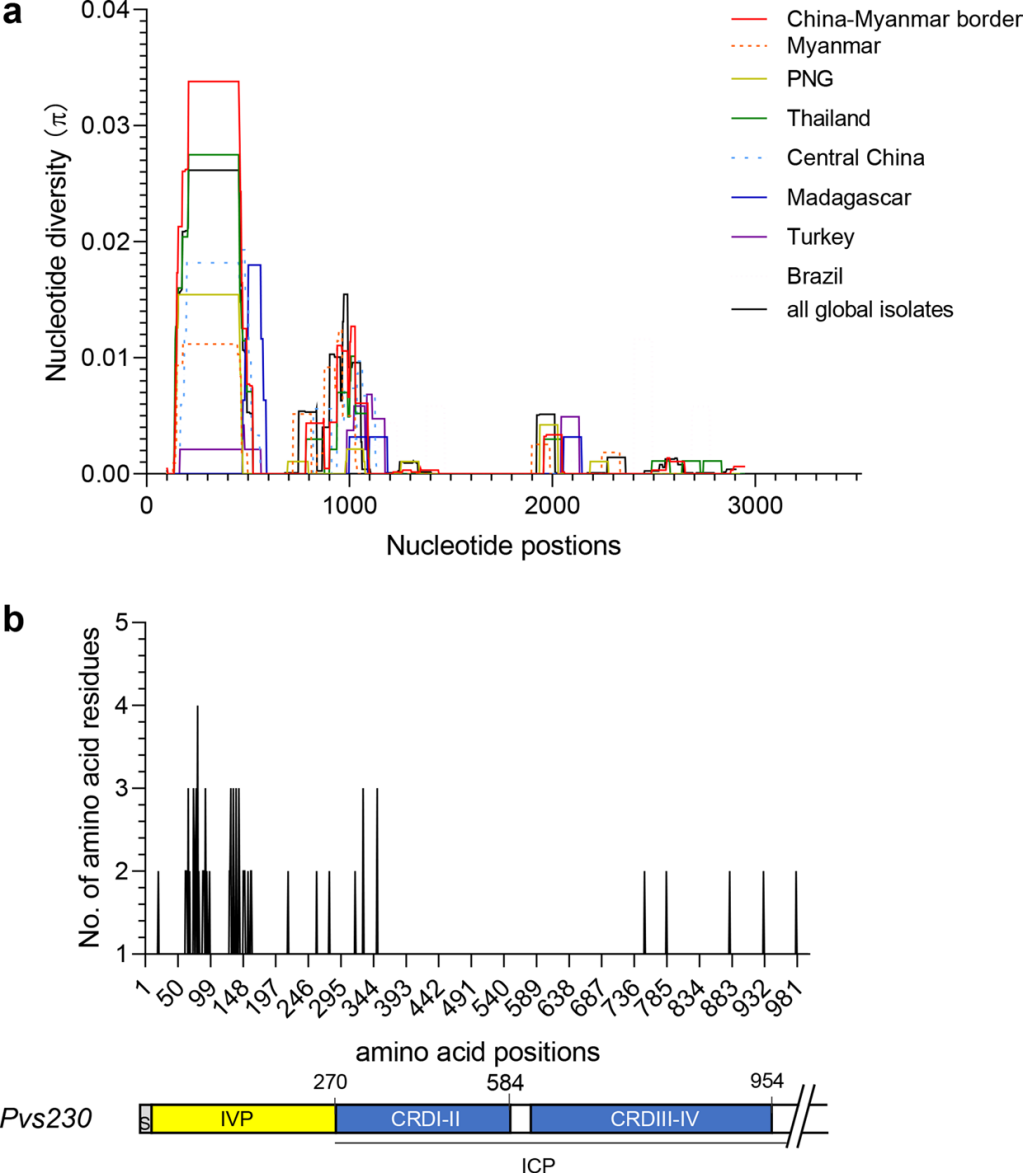
Nucleotide diversity (**a**) and amino acid polymorphism (**b**) of *Pvs230* from global isolates. A schematic of the IVP and ICP of Pvs230 is shown below. Nucleotide and amino acid positions refer to the Sal-I isolate sequence for *Pvs230*. CRD, cysteine-rich domain

The *Pvs230* gene was successfully amplified and sequenced from 166 *P. vivax* samples collected in the CMB area (*n* = 143) and central Myanmar (*n* = 23). For the sequenced region (nt 52–2862), there were 30 and 19 polymorphic sites in isolates from the CMB and Myanmar, respectively (Table 1). Of these, 14 and 13 polymorphic sites were in the IVP (nt 52–808), and 16 and 6 polymorphic sites were in the ICP (CRDI–IV, 808–2862 bp), respectively (Table 1). The average number of pairwise nucleotide differences (*k*) of the CMB isolates for the entire sequence, IVP, and ICP were 5.558, 3.518, and 2.041, respectively (Table 1). In comparison, the *k* values for these *Pvs230* fragments of the central Myanmar isolates were lower, at 3.553, 2.071, and 1.482, respectively. Nucleotide diversity (*π*) for the entire sequence, IVP, and ICP of *Pvs230* among the 143 *P. vivax* isolates of the CMB isolates were 0.002, 0.006, and 0.001, respectively (Table 1). For the Myanmar isolates, these values were 0.001, 0.004, and 0.001. A sliding window plot of *π* revealed values ranging from 0.000 to 0.023 for the CMB isolates and 0.000 to 0.016 for the Myanmar isolates (Fig. 1a). The CMB isolates had 47 *Pvs230* haplotypes, with an overall Hd of 0.935 (Table 1). The 23 Myanmar isolates had 15 haplotypes, with an Hd value of 0.925 (Table 1).

**Table 1:** Estimation of nucleotide diversity and summary statistics of *Pvs230* in 143 China–Myanmar border and 23 Myanmar isolates

Locality	Region	*S*	*k*	*π* ± SD	*H*	Hd ± SD	*d*_N_–*d*_S_	TD	D* (F&L)	F* (F&L)	MK
China–Myanmar border	Entire gene	30	5.558	0.002 ± 0.000	47	0.935 ± 0.009	0.002 ± 0.001*	0.076	−0.561	−0.365	6.410***
	IVP	14	3.518	0.006 ± 0.000	21	0.885 ± 0.012	0.010 ± 0.003**	1.021	0.331	0.702	7.233*
	ICP (CRDI–IV)	16	2.041	0.001 ± 0.000	27	0.813 ± 0.026	−0.000 ± 0.001**	−0.786	−1.161	−1.221	3.887
Myanmar	Entire gene	19	3.553	0.001 ± 0.000	15	0.925 ± 0.042	0.001 ± 0.001*	−1.134	−1.566	−1.611	2.096
	IVP	13	2.071	0.004 ± 0.001	8	0.751 ± 0.083	0.005 ± 0.002**	−1.444	−2.147	−2.257	4.626
	ICP (CRDI–IV)	6	1.482	0.001 ± 0.000	9	0.794 ± 0.065	−0.000 ± 0.001	−0.270	−0.219	−0.270	1.731

The total sequenced region included codons 18 to 957, IVP codons 1 to 269, ICP (CRDI–IV) codons 270 to 954. *S*, number of polymorphic (segregating) sites; *k*, the average number of nucleotide differences; *π*, pairwise nucleotide diversity; *H*, number of haplotypes; Hd, haplotype diversity; *d*_N_/*d*_S_, the ratio of non-synonymous to synonymous mutations; TD, Tajima’s *D* test; D* (F&L), Fu and Li’s D* value; F* (F&L), Fu and Li’s F* value; MK, McDonald–Kreitman test; SD, standard deviation

*P < 0.05; ***P < 0.001

The *Pvs230* sequences obtained from this study were compared with published sequences from six other geographical regions. A total of 48 mutations were identified among the 274 global and reference *Pvs230* sequences, including 16 synonymous SNPs (*SP* SNPs) and 32 non-synonymous SNPs (*NS* SNPs) (Table 2). The majority of the *NS* SNPs (17 sites, 53.1%) were clustered within the IVP, resulting in a peak *π* of 0.026 for this region (Fig. 1a). A total of 92 haplotypes were identified among the global isolates, demonstrating a high level of haplotype diversity (Hd = 0.968). Nucleotide diversity was 0.002 for the global samples, higher in East/Southeast Asia and Africa than in South America and Oceania, and the lowest in West Asia (Turkey) (Table 2). Except for the CMB and Thailand isolates, the *π* value in each population was lower than that in the global samples, suggesting country-specific substitutions. A sliding window plot of *Pvs230* in the global isolates revealed a peak *π* value of 0.026 at nt 207–479 of the IVP (Fig. 1a), including 10 of 46 amino acid changes (Fig. 1b).

**Table 2:** Genetic diversity of *Pvs230* among global isolates

Region	Country	*n*	*S*	*η*	*k*	*π* ± SD	NS	SP	*H*	Hd ± SD	GenBank accession numbers
East Asia	China–Myanmar	143	30	31	5.558	0.002 ± 0.000	22	9	47	0.935 ± 0.009	
	Central China	20	14	14	3.711	0.001 ± 0.000	12	2	11	0.911 ± 0.042	AB574574–AB574555
Southeast Asia	Myanmar	23	19	20	3.553	0.001 ± 0.000	17	3	15	0.925 ± 0.042	
	Thailand	20	19	20	4.411	0.002 ± 0.000	16	4	15	0.968 ± 0.025	AB574575–AB574594
West Asia	Turkey	20	6	7	1.774	0.001 ± 0.000	5	2	5	0.511 ± 0.128	AB574528–AB574547
Africa	Madagascar	7	7	7	2.762	0.001 ± 0.000	5	2	6	0.952 ± 0.096	AB574548–AB574554
South America	Brazil	20	8	8	3.079	0.001 ± 0.000	6	2	6	0.768 ± 0.062	AB574508–AB574527
Oceania	PNG	21	12	12	2.248	0.001 ± 0.000	9	3	9	0.767 ± 0.090	AB574595–AB574615
	Total	274	46	48	5.541	0.002 ± 0.000	32	16	92	0.968 ± 0.004	

*n*, number of isolates; *S*, number of polymorphic (segregating) sites; *η*, the total number of mutations; *k*, the average number of nucleotide differences; *π*, nucleotide diversity; NS, number of sites with non-synonymous polymorphisms; SP, number of sites with synonymous polymorphisms; *H*, number of haplotypes; Hd, haplotype diversity; SD, standard deviation; PNG, Papua New Guinea population

**Fig. 2:**
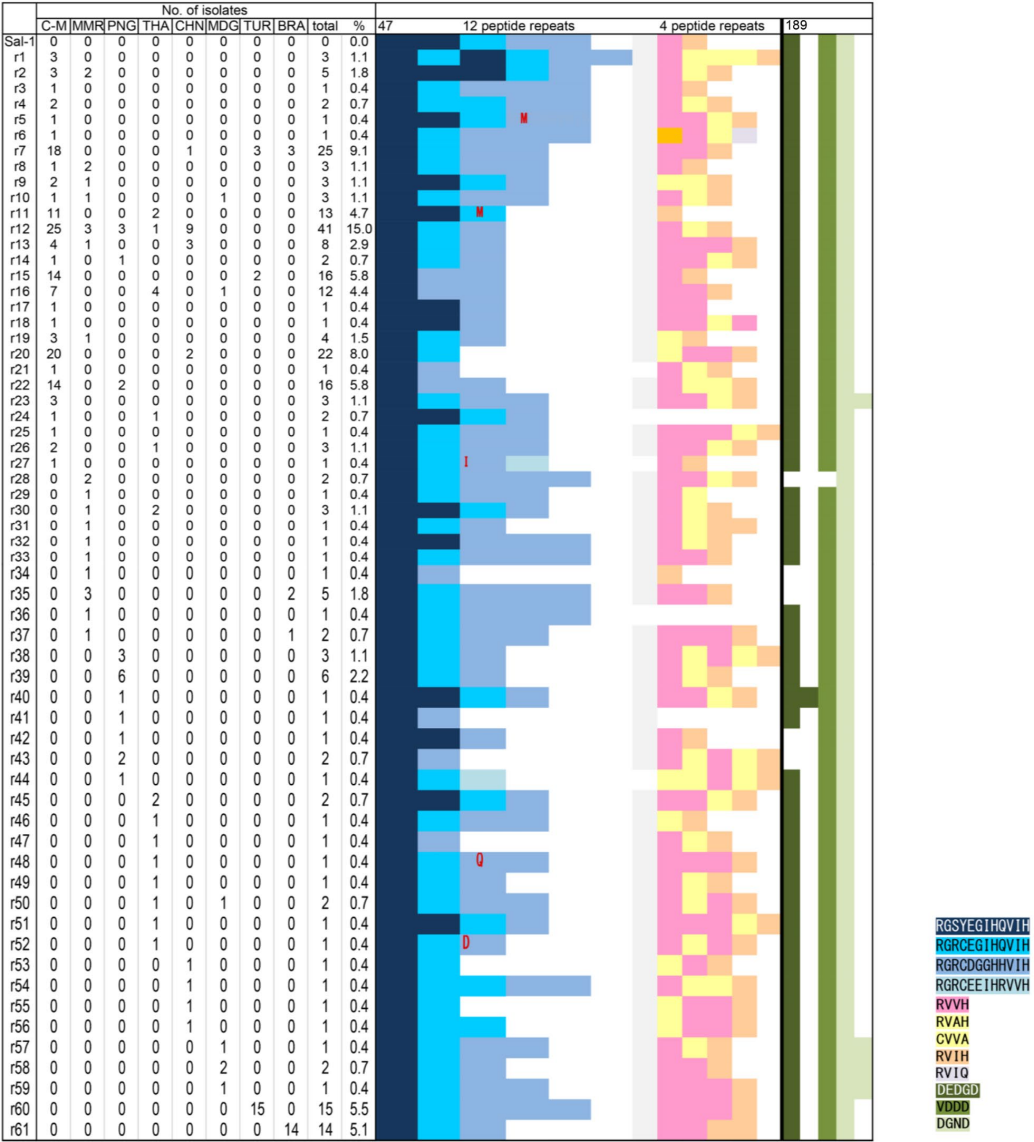
The pattern of the N-terminal repeat region in IVP of *Pvs230* from global isolates. Amino acid positions above the table are numbered after the Pvs230 sequence of the reference Sal-I strain. C–M, China–Myanmar border; MMR, Myanmar; PNG, Papua New Guinea; THA, Thailand; CHN, Central China; MDG, Madagascar; TUR, Turkey; BRA, Brazil

Two repeat sequences (RGXXXGXHXVIH and RVVH/RVIH/CVVA/RVIQ/RDVH) and degenerative repeats (DEDGD and DGND) were detected in the IVP of *Pvs230* (Fig. 2 and Additional file 2: Table S2). The number of repeats was variable among isolates: 2–5 times for the RGXXXGXHXVIH motif, and 0–5 times for the RVVH/RVIH/CVVA/RVIQ/RDVH motif in the CMB and Myanmar isolates. The absence of the RVVH/RVIH/CVVA/RVIQ/RDVH motif was only observed in Myanmar, Thailand, and PNG isolates. Variation in the number of the second repeat DEDGD–VDDD–DGND was limited and geographically restricted. One DEDGD was observed in all countries except Myanmar and PNG, where the repeat number was 0–2 (Fig. 2 and Additional file 2: Table S2). In addition, 1–2 repeats of DGND were observed in the CMB and Madagascar isolates.

### Evidence of positive diversifying selection on *Pvs230* gene

**Fig. 3:**
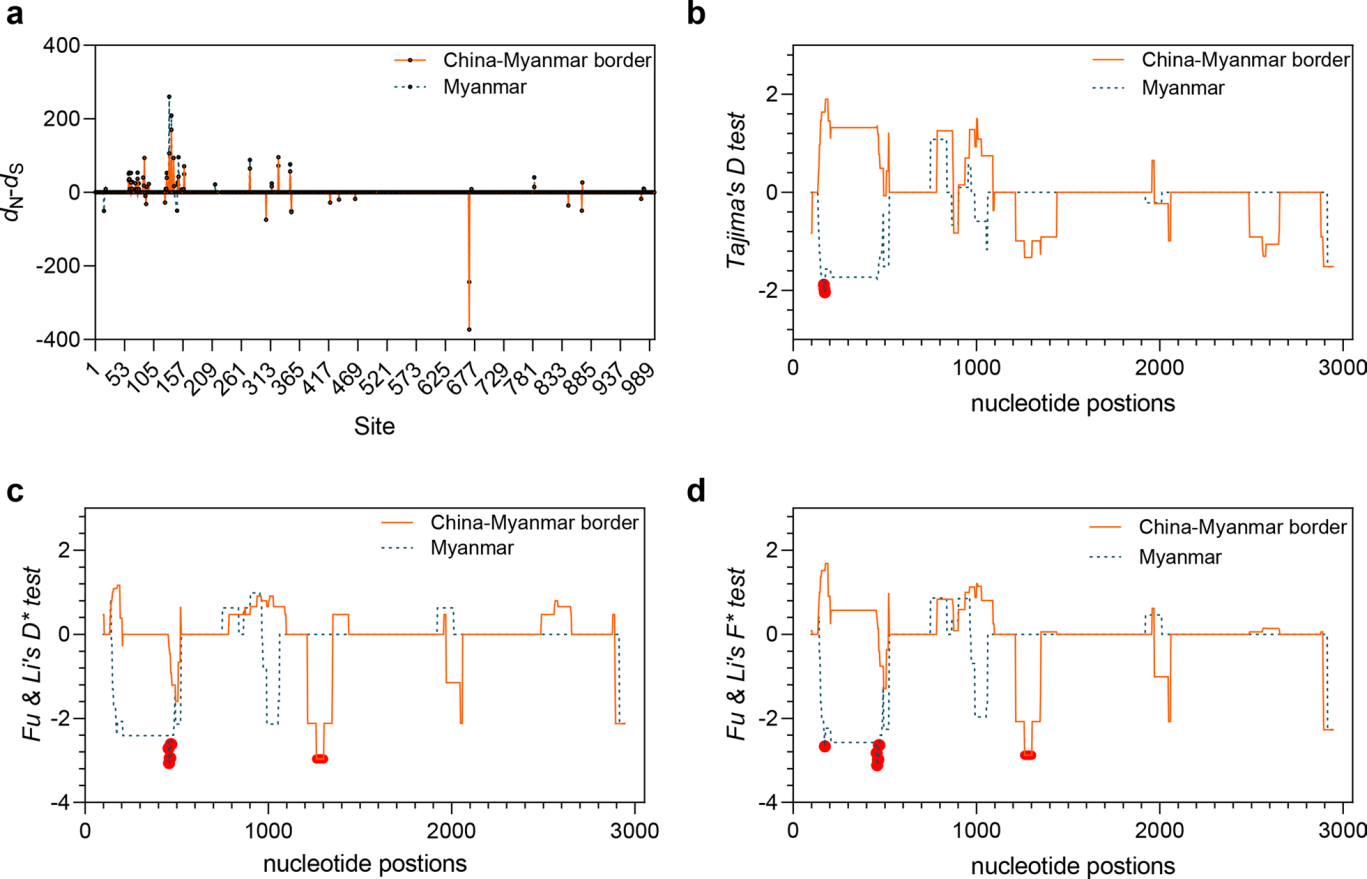
Natural selection on *Pvs230* in isolates from China–Myanmar border and Myanmar populations. Sliding-window plots of *d*_N_–*d*_S_ (**a**), Tajima’s *D* test (**b**), Fu and Li’s D* (**c**), and F* (**d**) tests for *Pvs230* are shown (window length = 90 bp and step size = 3 bp). Nucleotide numbers are based on the Sal-I *Pvs230* reference sequence. Red dots indicate regions with positive values (*P* < 0.05, one-tailed)

To examine whether natural selection contributed to the generation of the diversity in *Pvs230* of the CMB and Myanmar isolates, *d*_N_–*d*_S_, Tajima’s *D*, Fu and Li’s D* and F*, and MK test were performed. Although the values of Tajima’s *D*, and D* and F* were not significant for either the CMB or Myanmar isolates, differences in *Pvs230* domains existed, including positive values in the IVP for the CMB isolates and negative values in the ICP in both regions. Significant values of *d*_N_ over *d*_S_ were observed in the entire *Pvs230* sequence and IVP in isolates from both regions, suggesting positive diversifying selection. Sliding window plots depicted significant negative *D*, D*, and F* values in the IVP of Myanmar isolates. Meanwhile, significant negative D* and F* test values were detected in the ICP of CMB isolates, supporting purifying selection acting on this domain (Fig. 3). These observations were further supported by the SLAC and FUBAR tests identifying purifying selection on site 627, positive selection on 287 and 308 in the ICP of the CMB isolates, and positive selection on 287 in the Myanmar isolates (Additional file 3: Table S3). The MK test of the CMB isolates showed that the entire sequenced region (neutrality index [NI] = 6.410, *P* < 0.001), particularly within IVP (7.233, *P* < 0.05), had significantly more non-synonymous than synonymous substitutions expected from the comparison with *P. cynomolgi*. A similar pattern from the MK test was observed in the Myanmar isolates (Table 1), although the values were not significant. Together, these results suggest that polymorphisms found for *Pvs230*, especially in the IVP, are maintained by diversifying selection, presumably due to host immune pressure.

### *F*_ST_ analysis

*Plasmodium vivax* population differentiation was evaluated using the *F*_ST_ test. Pairwise population comparisons showed the highest level of genetic differentiation (*F*_ST_ = 0.609) between the West Asia (Turkey) and the Oceania (PNG) populations. High *F*_ST_ values (0.261–0.609) were detected between the East Asia (Central China) and Southeast Asia (Myanmar) populations, between West Asia and Brazil and other Asia populations, and between Oceania with Asia (Thailand, Myanmar, Turkey), Africa, and Brazil populations. A moderate range of *F*_ST_ values (0.173–0.249) were detected when comparing the CMB Myanmar, Oceania, and Africa populations with the Myanmar and West Asia populations, and with South America, Thailand, and Africa populations. *F*_ST_ values among East and Southeast Asia populations approached zero, indicating a high degree of genetic similarity. The lowest *F*_ST_ value (0.038) was observed between CMB and Thailand populations, suggesting extensive gene flow between these populations (Table 3).

**Table 3:** Genetic differentiation (*F*_ST_) of the *Pv230* gene among eight geographically different populations

Locality (no.)	China–Myanmar	Myanmar	PNG	Thailand	Central China	Madagascar	Turkey
China–Myanmar							
Myanmar	0.173***						
PNG	0.184***	0.361***					
Thailand	0.038**	0.119**	0.261***				
Central China	0.018	0.264***	0.187***	0.117**			
Madagascar	0.122***	0.144*	0.343***	0.089	0.217***		
Turkey	0.410***	0.178***	0.609***	0.432***	0.558***	0.486***	
Brazil	0.111***	0.249***	0.300***	0.179***	0.146**	0.196	0.503***

PNG, Papua New Guinea population

*P < 0.05; **P < 0.01; ***P < 0.001

### Genetic relationships between global isolates

**Fig. 4:**
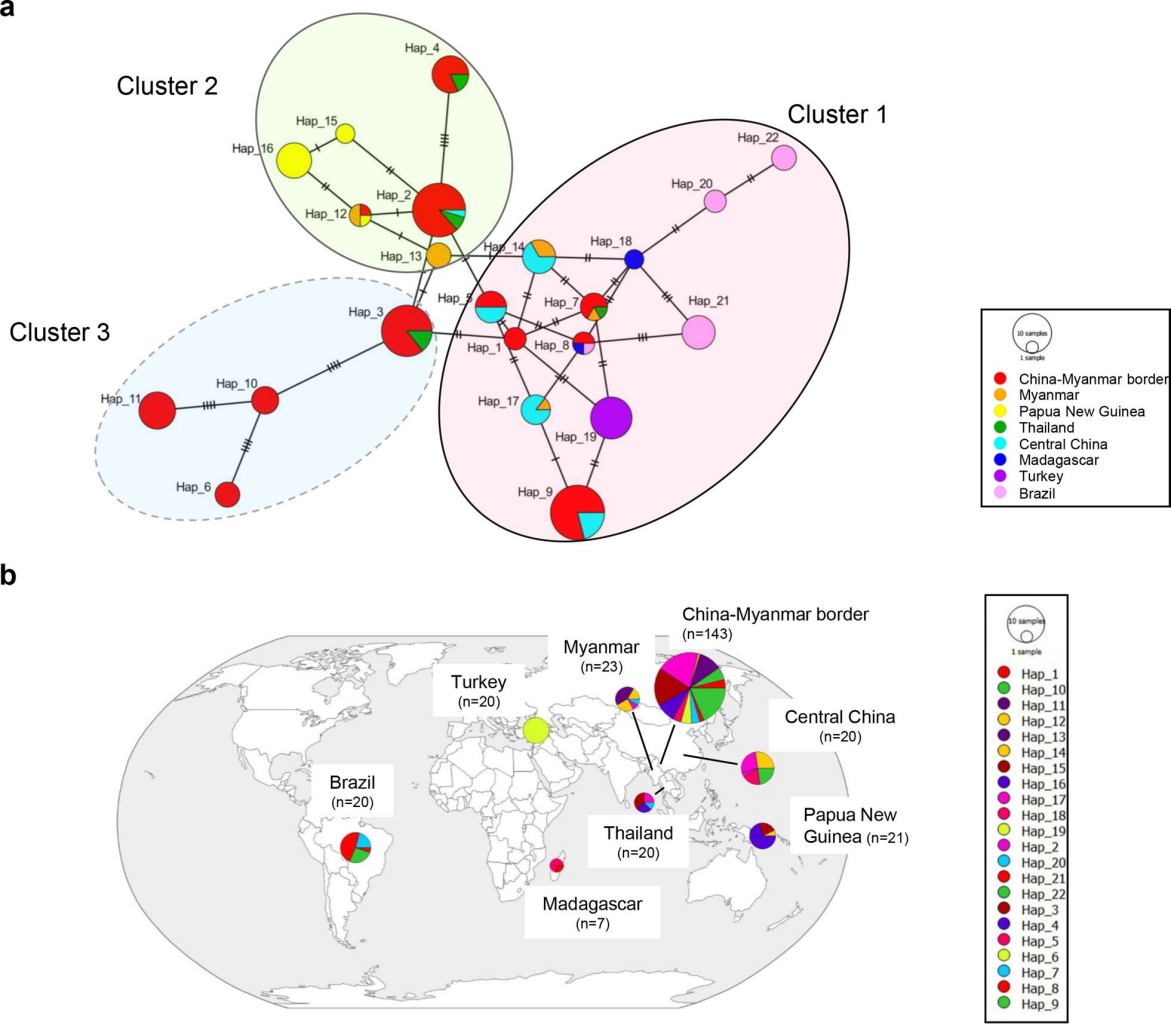
Haplotype network of *Pvs230* between eight geographically diverse populations. **a** Network analysis of *Pvs230* haplotypes. Haplotype networks were constructed using the median-joining algorithm implemented in PopArt software. Each node size reflects the frequency of a particular haplotype, and node color corresponds to the country origin. The lengths of the lines are proportional to genetic distance. **b** Worldwide distribution of *Pvs230* haplotypes. Sample size and origin are indicated

**Fig. 5:**
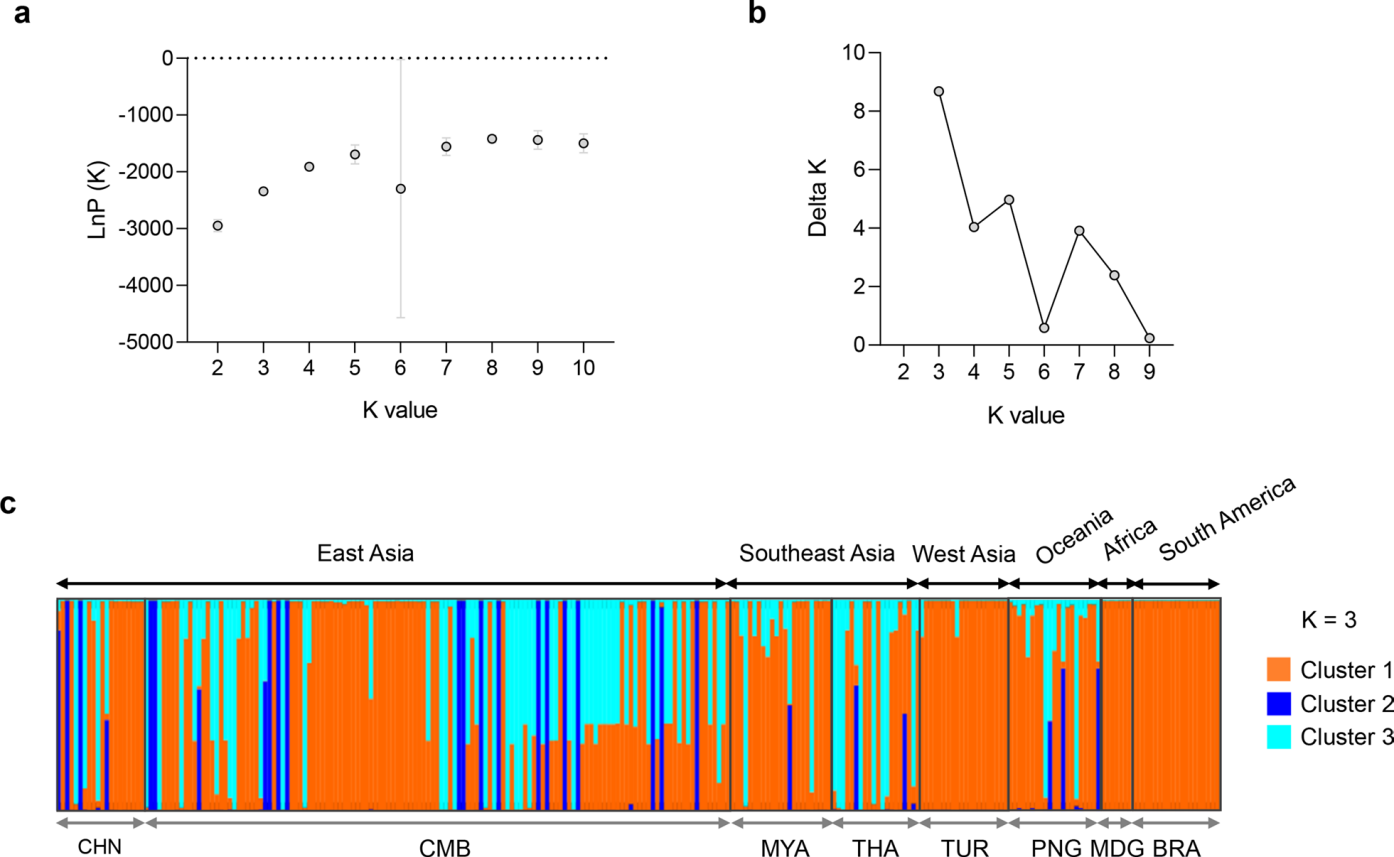
Clustering patterns of *Pvs230* haplotypes. **a** LnP(*K*) plot. **b** Delta *K* plot. **c** Clustering patterns of the *Pvs230* haplotypes (*K* = 3)

A total of 92 *Pvs230* haplotypes were identified in 274 global isolates, demonstrating a high level of haplotype diversity across the eight analyzed populations (Hd = 0.968). Of the 92 haplotypes, 63% (58/92) were single, and 84.8% (78/92) were region-specific. There was also significant haplotype sharing between regions and continents (Fig. 4a and b). For example, haplotypes 2, 3, 4, 5, 7, and 9 were shared between East and Southeast Asia populations. Haplotype 8 was the only haplotype present in three continents (Fig. 4a and b). The haplotype network showed three clusters, consistent with the phylogenetic analysis (Additional file 4: Fig. S1). The NJ tree revealed geographical clustering by countries or regions, with the Madagascar, Turkey, and Brazil haplotypes only observed in Group I. Group II consisted of admixed haplotypes from Oceania and three adjacent Asia populations (CMB, Myanmar, and Thailand), while 97.4% (37/38) of group III haplotypes were from CMB, Myanmar, Thailand, and Central China populations (Additional file 4: Fig. S1). Similar to the phylogenetic results, the STRUCTURE plot showed that 274 isolates from the eight global populations were grouped into three clusters (Fig. 5a, b). The parasite populations from the global isolates had admixed haplotypes, except for Madagascar and Brazil, where haplotypes were classified into only one cluster (Fig. 5c).

Evaluating the haplotypes within the ICP revealed 44 haplotypes from 274 global isolates, of which 45.5% were single (Additional file 5: Fig. S2). Individual haplotype prevalence ranged from 0.36 to 24.7%. There were region-specific haplotypes (e.g., haplotypes 1, 19, and 20 in Brazil). Haplotypes 4 and 5 were shared among the GMS populations. There was also haplotype sharing among continents (e.g., haplotypes 10, 11, and 14).

### Analysis of *Pvs230* structure and antigenicity

**Fig. 6:**
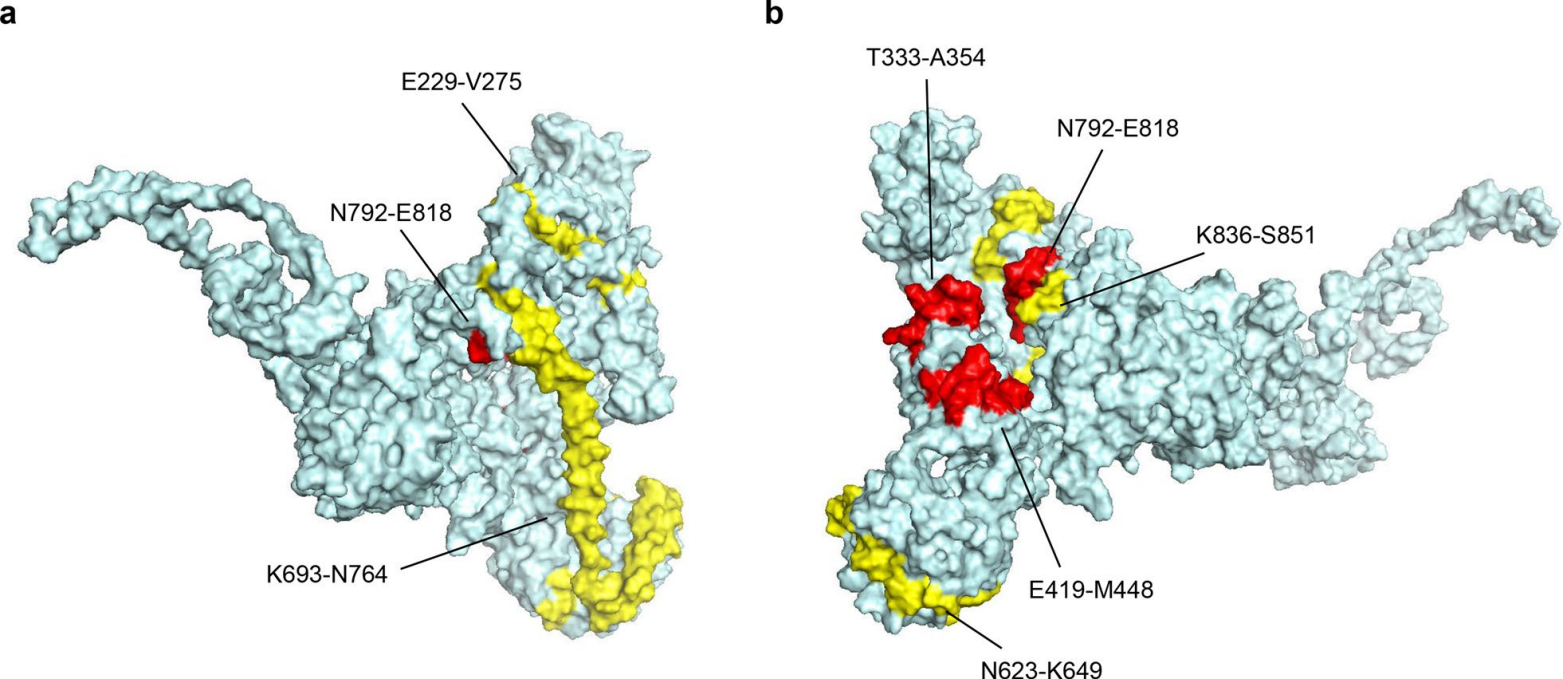
Predicted B-cell epitopes mapped on a 3D model of the Pvs230 protein. The opposing sides of the 3D structure are shown in (**a**) and (**b**). Overlapped regions of B-cell epitopes from two online prediction software programs with polymorphic resides (E229–V275, N623–K649, K693–N764, and K836–S851) are shown in yellow, and conserved overlapped regions (T333–A354, E419–M448, and N792–E818) are shown in red

The modeled structure of *Pvs230* was used to determine the distribution of the B-cell epitopes and the polymorphic residues. Six predicted potential B-cell epitopes were identified in the ICP of Pvs230 (Fig. 6 and Table 4). The overlap region of the putative B-cell epitopes from two prediction software algorithms is illustrated in the deduced 3D model shown in red (with polymorphic residues, N623–K649, K693–N764, and K836–S851) and yellow (without polymorphic residues, T333–A354, E419–M448, and N792–E818), respectively (Fig. 6). A moderate-level nucleotide and haplotype diversity was found in the B-cell epitopes E229–V275 (*π* = 0.006 ± 0.000, Hd = 0.590 ± 0.024) and N623–K649 (*π* = 0.006 ± 0.000, Hd = 0.457 ± 0.018), which contained two (V236F and V275M) and one (E631K) polymorphic residue, respectively. Lower nucleotide and haplotype diversity was found in epitopes E419–M448, K693–N764, N792–E818, and K836–S851, while the T333–A354 epitope was conserved, suggesting functional constraints of these regions.

**Table 4:** Polymorphisms in B-cell epitopes of the *Pvs230* gene from global isolates

No	AA position	Sequence	***S***	***NS***	***SP***	**π ± SD**		***H***	***Hd* ± SD**	***d***_**N**_***–d***_**S**_
1	E229–V275	EAGASDGVFDKVDEAFETTIKGDGNVLQASDPEVETFASSNTNKEYV	3	2	1	0.006 ± 0.000		6	0.590 ± 0.024	0.007
2	T333–A354	TLVNANEGSEEKKESLKEKRLA	0	0	0	0.000 ± 0.000		1	0.000 ± 0.000	0
3	E419–M448	EPYGQSVKGCNYTGKGKHFFSYDYEEGADM	1	0	1	0.000 ± 0.000		2	0.015 ± 0.010	− 0.001
4	N623–K649	NKAKYANLEMIPKMLKEEKKKKKNVLK	2	1	1	0.006 ± 0.000		3	0.457 ± 0.018	− 0.032
5	K693–N764	KKNSLFKNHKDSSYFDEVASPSDGFVKLLSFLDAQDTVILNEQVSDLTISTEQSTEDLFTLQLQIPPYITTN	3	3	0	0.001 ± 0.000		3	0.071 ± 0.021	0.001
6	N792–E818	NEHLVKGCNFSDGKMEHFTNNVGSTGE	1	0	1	0.000 ± 0.000		2	0.022 ± 0.012	− 0.001
7	K836–S851	KTVLPPSDEAEDSATS	1	1	0	0.001 ± 0.000		2	0.064 ± 0.020	0.002

*S*, number of polymorphic (segregating) sites; NS, number of sites with non-synonymous polymorphisms; SP, number of sites with synonymous polymorphisms; *π*, nucleotide diversity; *H*, number of haplotypes; Hd, haplotype diversity; SD, standard deviation

## Discussion

Due to the surface location and functional essentiality in forming exflagellation centers, Pvs230 has been considered a malaria TBV candidate and has undergone extensive studies [46, 47, 48]. The N-terminal tandem repeats of *Pvs230* are species-specific in *Plasmodium*. In this study, we found that the presence and extension of the N-terminal tandem repeats in the IVP of *Pvs230* differed by geographical origin, with most repeated patterns restricted to the region of origin. We found that replicate 12 (r12) was the most prevalent variant in the CMB isolates and was shared with Central China, Southeast Asia, and Oceania populations. Tandem repeats in some malaria vaccine candidate genes, such as circumsporozoite protein (CSP), differ according to transmission intensity and show an association with the response to treatment [49, 50, 51]. The function for the N-terminal repeats in *Pvs230* of *P. vivax* remains elusive.

Polymorphic sites were distributed unevenly across *Pvs230*, with most variations accumulated in IVP and CRDI–II in ICP, suggesting that these regions are potentially more exposed to the host immune system. Immunological studies indicate that the pro-domain (downstream of the cleavage site in IVP) and CRDI–II domain in ICP could elevate a naturally acquired antibody response in endemic areas [11, 52, 53]. Evidence showed that antibodies against the Pvs230 ICP exhibit a profound inhibitory effect on oocyst formation [18]. Recombinant Pvs230 proteins with varying CRD domain combinations showed different TBA, with antibodies targeting the CRDI domain triggering the strongest TB activity, suggesting that this domain is a promising target for TBV development [54]. In our study, several non-synonymous substitutions under positive selection, including A287D/T and L308I/V, were identified in the CRDI domain in the CMB and Myanmar isolates. Whether these substitutions will affect the efficiency of a TBV that targets these regions needs further experimental examination. This study identified 92 *Pvs230* haplotypes in the global isolates, with varying levels of haplotype diversity among regions. The highest diversity was detected in Asia, where 73 haplotypes were reported, including the two major haplotypes (2 and 9) and several minor haplotypes, indicating a close genetic relatedness between these *P. vivax* populations, comparable to the analysis of *Pvhap2* sequences [55]. The lowest haplotype diversity was observed in Turkey, indicating more genetically related parasite isolates.

*Pv230* showed low genetic diversity (*π* = 0.001–0.002), consistent with a previous report [23]. This result is also comparable to the low genetic diversity observed for other TBV candidates reported in *P. vivax*, including *Pvs28/25* (*π* = 0.0024/0.0017, Asia; *π* = 0.0024/0.0018, Americas; [56]), *Pvs48/45* (*π* = 0.00173, global isolates, [45]), *Pvhap2* (*π* = 0.0002, CMB; *π* = 0.0005, Myanmar, [55]), and *Pvceltos* (*π* = 0.001, Brazil [57]). This high level of conservation may be a consequence of the functional constraint of these proteins in sexual development. Neutrality tests including *d*_N_–*d*_S_ and the MK test detected positive selection in the IVP, which was further supported by the codon-based selection tests, indicating that the IVP may be targeted by the host immune system. The N-terminal region upstream of the proteolytic cleavage site of Pfs230 is released into the medium after processing during gametogenesis, and might be important for immune evasion [58]. Pfs230 protein sequences downstream of the cleavage site in the IVP could elicit TBA comparable to that in the ICP [16]. Although the cleavage site is not conserved between Pfs230 and Pvs230, the evolutionarily conserved feature of predicted B-cell epitopes mapped around this region (E299–V275) may be beneficial for TBV design. In contrast, significant values of *d*_s_ over *d*_N_ in ICP of the *pvs230* gene from both the CMB and Myanmar isolates suggest that purifying selection acts on this region. Earlier studies showed that an intact ICP is important for the gamete surface localization of Pfs230 [59], and the CRDI within ICP is critical for the generation of an effective antibody with TBA [15, 18, 60, 61], suggesting that this region may be critical for the gamete fertilization function of Pfs230, and could be a promising target for TBV development. We identified several non-synonymous substitutions in the B-cell epitopes predicted in the ICP. The codon-based test for selection also revealed two sites (287 and 308) under positive selection in the CRDI domain in the CMB isolates. Therefore, future investigations are needed to determine whether these mutations could interfere with TBV efficiency.

Despite low sequence diversity, *Pvs230* showed divergent haplotype distribution patterns in different *P. vivax* populations. Population differentiation, especially among the Asian populations, was supported by both Wright’s *F*_ST_ statistic and phylogenetic analysis, which indicated population subdivision among the *P. vivax* populations. The *F*_ST_ estimation between East/Southeast Asia (Central China, CMB, and Thailand) and West Asia (Turkey) populations was remarkable (0.41–0.558), whereas parasites from the GMS exhibited only slight geographical genetic differentiation. Although antigens under selection may not be suitable markers for population genetic studies, the results from *Pvs230* analysis are highly congruent with the population genomics studies conducted using whole genome sequencing data from the GMS [62]. These less differentiated *P. vivax* populations suggest the lack of gene flow barriers in the GMS, which may be further enhanced by the extensive human migration in this region [63, 64]. In addition, although the world *P. vivax* populations were grouped into three clusters by structure, phylogenetic, and haplotype network analyses, similar haplotypes in the *Pvs230* sequences were found in many parts of the world. Therefore, geographical differentiation of the parasite population and conservation of certain *Pvs230* haplotypes should be considered for Pv230-based vaccine design.

## Conclusions

This study revealed limited genetic diversity in the *Pvs230* gene, and neutrality tests showed that the gene is under positive selection on the IVP and under purifying selection on the ICP. The study revealed continent-specific and shared *Pvs230* haplotypes in the global isolates, and further reinforced the observation from genomic analysis, showing little genetic differentiation in the GMS. The presence of dominant haplotypes in the ICP circulating in East and Southeast Asia parasite populations suggests the possibility of developing a universal Pvs230-based TBV in this region.

## Supplementary Information

**Additional file 1: Table S1.** Primers used in *Pvs230* amplification and sequencing.

**Additional file 2: Table S2.** The diversity pattern of the N-terminal repeat region in IVP of *Pvs230* from global isolates. Related to Fig. 2.

**Additional file 3: Table S3.** Codon-based tests for selection on *Pvs230* in China–Myanmar border and Myanmar isolates.

**Additional file 4: Figure S1.** Phylogenetic analysis of *Pvs230* sequences from global *P. vivax* populations. The maximum-likelihood phylogenetic tree reconstructed based on alignment by ClustalW with bootstrap analysis to assess clade support (500 replicates) was shown for *Pvs230* global isolates. The global isolates were clustered into three main groups, the branches of which are shown in red (group 1), green (group 2), and blue (group 3).

**Additional file 5: Figure S2.** Median-joining network of *Pv230* among eight geographically diverse populations. Haplotypes composed of nucleotide polymorphism in ICP of *Pv230* with a frequency > 1 were used to create a median-joining network. Each node represents one haplotype, node size indicates haplotype frequency, and node color corresponds to the country of origin. Line length is proportional to genetic distance.

## Data Availability

The datasets supporting the conclusions of this article are included within the article.

## Abbreviations

TBV: Transmission-blocking vaccine
IVP: Interspecies variable part
ICP: Interspecies conserved part
NMCPs: National Malaria Control Programs
TBA: Transmission-blocking activity
SMFA: Standard membrane-feeding assay
MK: McDonald–Kreitman test
*F*_ST_: Fixation index
SP SNPs: Synonymous single-nucleotide polymorphisms
NS SNPs: Non-synonymous single-nucleotide polymorphisms
PNG: Papua New Guinea
MCMC: Markov chain Monte Carlo
3D: Three-dimensional
NI: Neutrality index
CSP: Circumsporozoite protein
