# Supplementary figures and images for: Genetic diversity in the transmission-blocking vaccine candidate Plasmodium vivax gametocyte protein Pvs230 from the China–Myanmar border area and central Myanmar
Source: Parasit Vectors. 2022 Oct 17;15:371. doi: 10.1186/s13071-022-05523-0 (PMC9574792; doi:10.1186/s13071-022-05523-0)

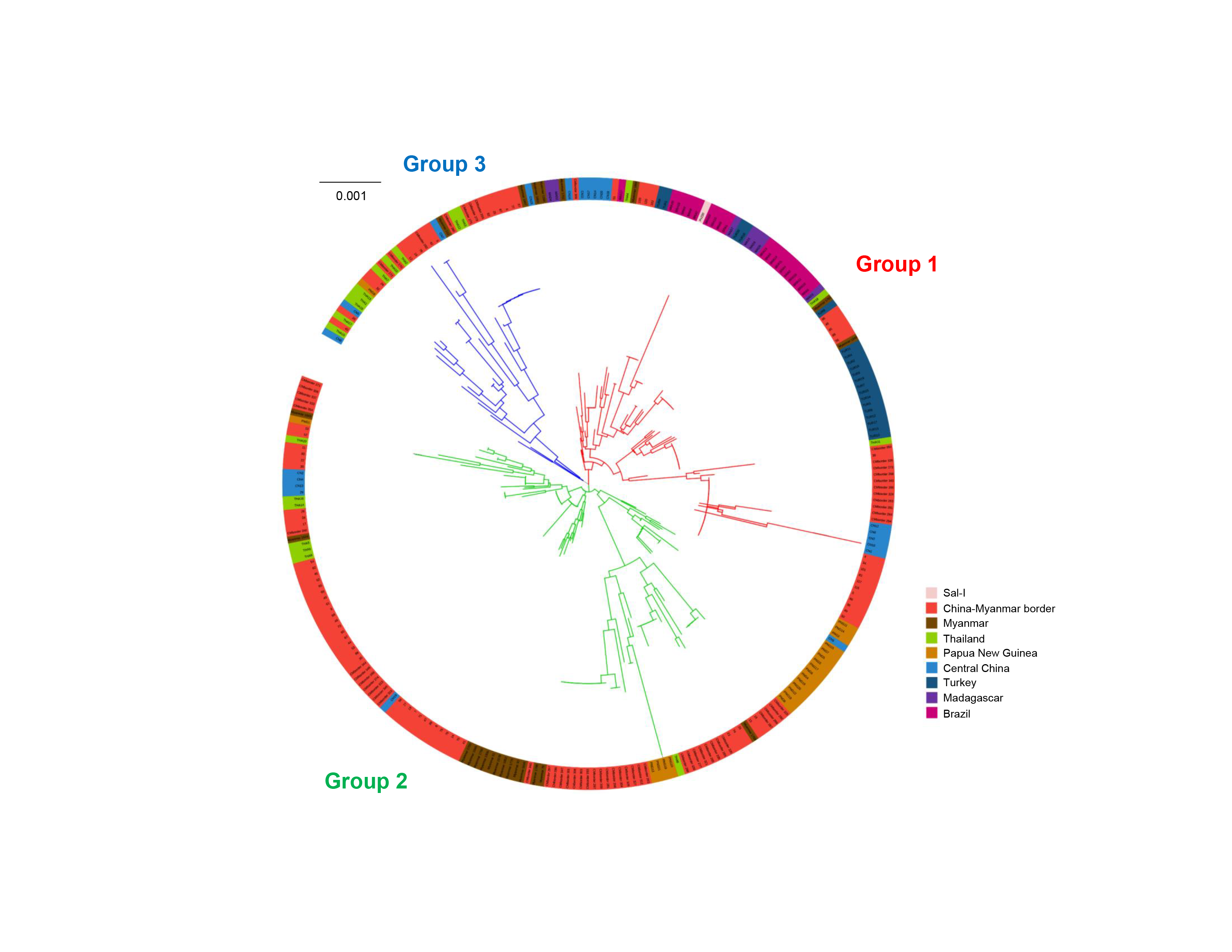

Supplement: Supplementary file 4 — Additional file 4: Figure S1. Phylogenetic analysis of Pvs230 sequences from global P. vivax populations. The maximum-likelihood phylogenetic tree reconstructed based on alignment by ClustalW with bootstrap analysis to assess clade support (500 replicates) was shown for Pvs230 global isolates. The global isolates were clustered into three main groups, the branches of which are shown in red (group 1), green (group 2), and blue (group 3). [file 13071_2022_5523_MOESM4_ESM.tif]

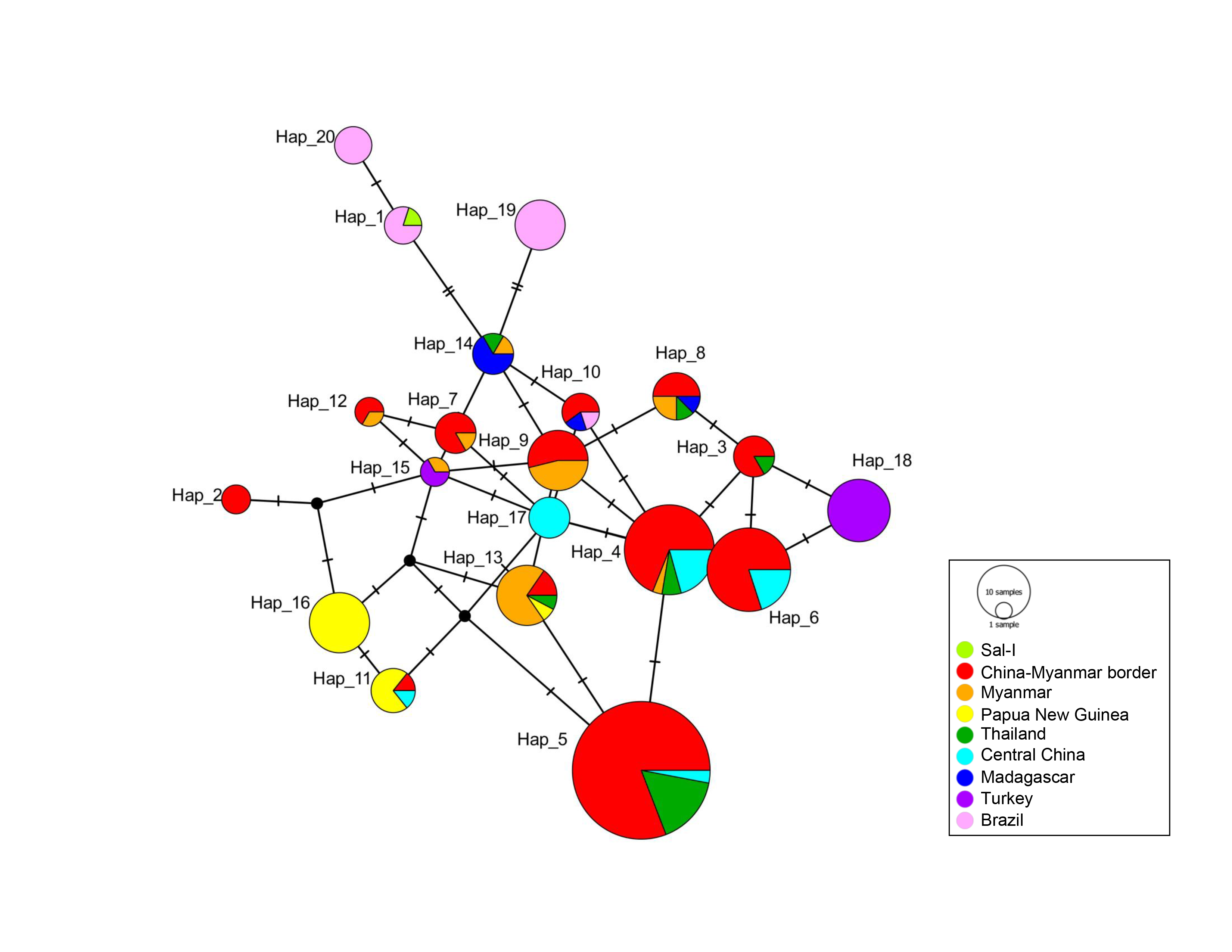

Supplement: Supplementary file 5 — Additional file 5: Figure S2. Median-joining network of Pv230 among eight geographically diverse populations. Haplotypes composed of nucleotide polymorphism in ICP of Pv230 with a frequency > 1 were used to create a median-joining network. Each node represents one haplotype, node size indicates haplotype frequency, and node color corresponds to the country of origin. Line length is proportional to genetic distance. [file 13071_2022_5523_MOESM5_ESM.tif]
